# Depletion of Brain Docosahexaenoic Acid Impairs Recovery from Traumatic Brain Injury

**DOI:** 10.1371/journal.pone.0086472

**Published:** 2014-01-27

**Authors:** Abhishek Desai, Karl Kevala, Hee-Yong Kim

**Affiliations:** Laboratory of Molecular Signaling, National Institute of Alcohol Abuse and Alcoholism, National Institutes of Health, Bethesda, Maryland, United States of America; University of Naples Federico II, Italy

## Abstract

Omega-3 fatty acids are crucial for proper development and function of the brain where docosahexaenoic acid (DHA), the primary omega-3 fatty acid in the brain, is retained avidly by the neuronal membranes. We investigated the effect of DHA depletion in the brain on the outcome of traumatic brain injury (TBI). Pregnant mice were put on an omega-3 fatty acid adequate or deficient diet from gestation day 14 and the pups were raised on the respective diets. Continuation of this dietary regime for three generations resulted in approximately 70% loss of DHA in the brain. Controlled cortical impact was delivered to both groups of mice to produce severe TBI and the functional recovery was compared. Compared to the omega-3 adequate mice, the DHA depleted mice exhibited significantly slower recovery from motor deficits evaluated by the rotarod and the beam walk tests. Furthermore, the DHA deficient mice showed greater anxiety-like behavior tested in the open field test as well as cognitive deficits evaluated by the novel object recognition test. The level of alpha spectrin II breakdown products, the markers of TBI, was significantly elevated in the deficient mouse cortices, indicating that the injury is greater in the deficient brains. This observation was further supported by the reduction of NeuN positive cells around the site of injury in the deficient mice, indicating exacerbated neuronal death after injury. These results suggest an important influence of the brain DHA status on TBI outcome.

## Introduction

Traumatic brain injury (TBI) is a leading cause of death and disability contributing to a third of all injury-related deaths in the USA [1]. Although people engaged in certain professions such as military personnel, amateur and professional athletes are at a higher risk of suffering from TBI, it is encountered in all populations. TBI can manifest across a wide range of responses, depending on the severity of injury. Mild concussion can lead to temporary state of confusion and transient unconsciousness while severe brain injury may result in loss of function of the limbs, speech impairment, disturbances in normal memory and emotional responses. Excitotoxicity, oxidative stress and inflammation are the primary mechanisms that lead to neuronal cell death and dysfunction in models of brain injury [2].

The polyunsaturated fatty acids linoleic (LA, 18:2n-6) and linolenic acid (LNA, 18:3n-3) are essential fatty acids that cannot be synthesized by the body. LNA serves as the precursor for long chain omega-3 fatty acids such as docosahexaenoic acid (DHA) while LA is converted into long chain omega-6 fatty acids such as arachidonic acid (AA) [3]. DHA and AA are abundantly found in the brain, where these are stored mainly in membrane phospholipids. Immediately after the injury, phospholipases are activated, leading to an increase in free fatty acids [4]. DHA has been shown to increase neurite outgrowth and synaptogenesis, and promotes glutamatergic neurotransmission through increase in glutamate receptor subunit expression [5]. DHA can directly interact with nuclear receptors such as retinoid acid X receptor, activating transcriptional activity [6]. Moreover, DHA has been shown to be converted to anti-inflammatory, proresolving and neuroprotective mediators, such as resolvins [7] and protectins [8]. It has been also demonstrated that DHA metabolizes to synaptamide that has neuritogenic/synaptogenic properties [9]. In contrast, AA is converted by cyclooxygenases into 2-series prostaglandins and 4-series leukotrienes, most of which exert pro-inflammatory effects [10]. Supplementation of DHA exerts neuroprotective effects and has been reported to afford protection from diffuse axonal injury [11] and mixed brain injury [12] as well as in Alzheimer’s disease model [13], cerebral ischemia [14], [15], and Parkinson’s disease [16], [17]. However, not much is known regarding the effect of pre-existing membrane levels of DHA on the outcome after cortical impact injury. In the present study, we have investigated the consequence of severe depletion of brain DHA on the behavioral and histological outcome of focal brain injury in a mouse model after manipulating the brain DHA status through multi-generational feeding with an omega-3 fatty acid deficient diet.

## Materials and Methods

### Animals and Diets

Pregnant E14 C57BL/6J mice procured from the Jackson Laboratory were placed on either omega-3 fatty acid adequate or deficient diet as described earlier [5]. The modified AIN-93G [18] diets with custom fat mixture were procured from Dyets Inc. (Bethlehem, PA, USA). The lipids in the omega-3 adequate diet consisted of tocopherol-stripped safflower oil (17.7 gm/kg), flaxseed oil (4.81 gm/kg), hydrogenated coconut oil (74.49 gm/kg) and alga-derived DHA oil (DHASCO; DSM-Martek Biosciences, Columbia, MD, USA) (3 gm/kg). The lipids in the omega-3 fatty acid deficient diet were tocopherol-stripped safflower oil (19 gm/kg) and hydrogenated coconut oil (81 gm/kg) (Table 1). The resulting fatty acid composition (%) of adequate vs. deficient diets was as following; LA, 13.8 vs. 13.8; LNA, 2.5 vs. 0.09; DHA, 0.9 vs. 0; and no AA or eicosapentaenoic acid (EPA) was detected. The litters were weaned on the same diet as their dams and considered the second generation on the adequate or deficient diet. They were mated around 3 months of age to produce the third generation pups that were again placed on the same diet after weaning as their dams. The nervous tissue of the third generation mice was substantially depleted of DHA. Mice from the third generation were used for the TBI experiments when they were 3-4 months old.

**Table 1:** Composition of omega-3 fatty acid adequate and omega-3 fatty acid deficient diets^1^.

	Adequate Diet		Deficient Diet	
	*gm/kg*	*kcal/gm*	*gm/kg*	*kcal/gm*
Vitamin Free Casein	200	744	200	744
L-Cystine	3	12	3	12
Cornstarch	150	540	150	540
Sucrose	100	400	100	400
Dextrose	199.5	726	199.5	726
Maltose Dextrin	150	570	150	570
Cellulose	50	0	50	0
Tocopherol Stripped Safflower Oil	17.7	159.3	19	171
Flaxseed Oil	4.8	43.3	0	0
Hydrogenated Coconut Oil	74.5	670.4	81	729
DHASCO^2^	3	0	0	0
Mineral Mix#210025	35	30.8	35	30.8
Vitamin Mix#310025	10	38.7	10	38.7
tBHQ^3^	0.02	0	0.02	0
Choline Bitartrate	2.5	0	2.5	0

1 These diets were prepared by Dyets Inc. (Bethlehem, PA, USA) based on AIN-93G (18) and have been used to achieve DHA depletion in rodents (24,36).

2 DHASCO: DHA Algal Oil.

3 tBHQ: tert-butylhydroquinone.

### TBI model

All experimental procedures were approved by the institutional Animal Care and Use Committee (LMS-HK-03) and the US Army Medical Research and Materiel Command (USAMRMC) Animal Care and Use Review Office (ACURO), and performed according to the NIH Guide for Care and Use of Laboratory Animals. TBI was inflicted by controlled cortical impact using the Head Impactor TBI 0310 (Precision Systems and Instrumentation, LLC), a pneumatically contolled impactor for precise injury. Each mouse was anaesthetized using 4% isofluorane and fixed onto a stereotaxic frame (David Kopf Instruments, Tujunga, CA, USA). The anesthesia was maintained with 2.5% isoflurane. A midline incision was made on the scalp and a 4 mm craniotomy was made over the left cerebral hemisphere between the lambda and the bregma about 1 mm from the midline. The stereotaxic frame was inclined so as to make the plane of the cortex perpendicular to the impactor tip. The impact velocity was set at 3.5 m/s with the penetration depth at 1.5 mm and the impactor dwell time was 500 ms. After the injury, the wound was covered with Surgicel (Johnson & Johnson, Arlington, TX, USA) and the craniotomy was closed using cyanoacrylate. The body temperature of the mouse was maintained around 36–37°C with an infra-red warmer throughout the surgical procedure. The operated mice were placed in cages over a warm water blanket after surgery and allowed to recover with free access to the same diet as before surgery. Sham operated mice received craniotomy without brain injury.

### Behavioral tests

Rotarod test. The rotarod test has been reported to be effective for evaluating motor deficits after TBI [19]. Rotarod apparatus from Med Associates (St. Albans, Vermont, USA) was used to assess the recovery in motor functions. Mice were trained on the accelerating rotarod for three days and scored on the fourth day, the day preceding the day of surgery. This score was considered the baseline Day 0 score. After surgery, mice were tested daily for six days. Each test session consisted of three trials separated by an interval of about ten minutes. The rotarod accelerated from 4 to 400 rotations per minute over the trial span of 5 min. The latency of mice to fall from the rotarod was recorded for each trial. The trial was also stopped after two consecutive passive rotations on the rotarod. The latency scores obtained from each trial were averaged.

Beam walk test. The beam walk test has been employed to assess fine motor coordination in rodents by counting the foot slips that occur while walking from one end of a narrow beam to the other [20]. A 50 cm long and 7 mm wide elevated plastic beam was used for this test. One training or test session was performed daily except on the day of surgery. Each daily session consisted of three trials, in which the number of hind-limb foot slips and the total number of steps were counted. The percent foot slips was calculated after adding the foot slips made and the steps taken in the three trials. Mice were trained to traverse the beam for two days and pre-surgery baseline performance was assessed on the third day. Daily assessments were made following the day of surgery.

Open field and novel object recognition tests. The novel object recognition test was used to assess the ability of mice to recognize and discriminate novel objects, which was taken as a measure of the memory of the familiar object [21]. From the fifth to the seventh day after TBI, the mice were allowed to explore an arena enclosed by a 40×4 cm open box made of black plexiglass (Stoelting, Wood Dale, Illinois, USA) for 5 min each day. The exploration of the arena for 5 min on the fifth day after surgery constituted the open field test. Following habituation on the seventh day, the mice were introduced into the arena which now had two identical objects. Each mouse was allowed to explore the two objects in the training period and the duration of exploration was monitored. The mouse was considered to explore an object if its nose was within 2 cm of the object and the mouse was sniffing or apparently focusing its attention on the object. This trial period was continued till the total object exploration time reached 30 seconds or for 15 min in the enclosure. One of the objects was replaced by a distinctly different object of comparable dimensions and the mice were individually re-introduced into the enclosure three hours after the trial. The time of exploration of the familiar and the novel object was monitored for 4 min.

### Fatty Acid Analysis

The tissues were homogenized in an ice cold mixture of 50/50 methanol/BHT (50 mg/L butylated hydroxytoluene) and buffer (15 mM tris, pH 7.4; 150 mM NaCl) with a Potter-Elvehjem homogenizer. Lipids were extracted by the method of Bligh and Dyer [22]. In brief CHCl3 and water were added to bring the CHCl_3_:CH_3_OH:H_2_O ratio in samples to 2:2:1.8, followed by vigorous vortexing under nitrogen. Samples were spun at 3,000 rpm at 4°C and the bottom organic layers transferred to clean glass tubes. The extraction was repeated twice with the remaining aqueous layer. Pooled organic fractions were dried under nitrogen and suspended in 2∶1 CHCl_3_:CH_3_OH. Fatty acids in the lipid extract were quantified by gas chromatography analysis after transmethylation with boron trifluoride/CH_3_OH, followed by hexane extraction as described previously [23].

### Western Blot Analysis

Brain cortical tissue of mice on omega-3 adequate and deficient diet groups was excised at 24 h and 7 days after TBI in two separate experiments. The α-spectrin cleavage was assessed at 24 h while synapsin 1 expression was assessed at 7 days after TBI. The tissue was homogenized manually in 2X lysis buffer (Cell Signaling Technology, Danvers, MA, USA) using a Potter-Elvehjem homogenizer, briefly sonicated and centrifuged to obtain the supernatant. Protein content was estimated and cortical samples were run in gradient polyacrylamide gels (Novex, Invitrogen, Carlsbad, CA, USA) and subsequently transferred onto a PVDF membrane (Millipore, Billerica, MA, USA). After blocking with 5% milk, mouse α-spectrin antibody (Santa Cruz Biotechnology, 1∶800) or synapsin-1 antibody (Sigma, 1∶1000) was used to probe for the respective protein, which were detected using Pierce supersignal west pico chemiluminescent detection substrate (Thermo Fisher Scientific, Rockford, IL, USA).

### NeuN Immunostaining

50 µm free floating brain sections were cut with a vibratome. Five alternate brain sections from each mouse from the epicenter of injury were used for NeuN staining. All the staining steps were performed on a rocker. The sections were washed with PBS and permeabilized with 0.2% Triton X in PBS for 2 h. They were then immersed in 1% bovine serum albumin (BSA) in 0.1% tween 20 in PBS for 1 h. The sections were then immersed in anti-NeuN mouse monoclonal antibody (Millipore, 1∶300) for 24 h on a rocker at 4°C. The sections were then washed with 0.1% tween 20-PBS thrice, immersed in Alexa-Fluor anti-mouse 555 (1∶200) for 24 h in the refrigerator, washed thrice and mounted with immunocruz mounting medium (Santa Cruz Biotechnology, Inc.). The NeuN positive cells were observed using a 20X objective in 800 µm peri-contusion area. The images were captured using an inverted motorized IX81 Olympus microscope. For each non-overlapping field, a series of ten images was captured along the Z axis at 2 µm intervals and NeuN positive cells were counted for six fields per section using Metamorph software.

### TBI lesion volume

For each brain, seven brain sections that were 400 µm apart and included the TBI lesion were mounted onto glass slides and stained with cresyl violet. The digital photograph of each 50 µm stained section was analyzed using ImageJ software (NIH, Bethesda, MD USA). The boundary of the lesion was carefully traced using the polygon tool and the lesion area was calculated. The lesion volume was then obtained by multiplying the sum of the lesioned area obtained from each section by the distance between the sections.

### Statistical analysis

Two-way AVOVA was used followed by Holm-Sidak’s multiple comparison test for the rotarod and beam walk tests with GraphPad Prism version 6.00 for Windows, GraphPad Software (La Jolla, California, USA). The densitometric data from western blots were analyzed by one-way ANOVA with Fishers LSD test. The statistical analyses of other tests were done by Students unpaired two-tailed *t*-test. Unless specified otherwise, all data were expressed as mean ± SEM.

## Results

### Brain Fatty Acid Analysis

To verify the effect of the respective diets on the brain levels of fatty acids, we assessed cerebellar lipid composition on Day 7 after TBI (Table 2). The fatty acids that were most affected by the two diets were DHA and docosapentaenoic acid (DPA n-6) (Table 1). There was more than three-fold decrease in the DHA levels (from 15.1±2.1 to 3.9±1.0%) in the brain of mice fed with omega-3 deficient diet. As expected, the omega-3 deficient diet significantly increases brain DPAn-6 levels, from the undetectable level to 7.7±0.7%. Thus the brains of the mice fed with omega-3 deficient diet for three generations had substantial depletion in DHA, which was replaced by DPA as has been described earlier [24].

**Table 2:** Fatty acid analysis using the cerebellum of TBI mice on omega-3 fatty acid adequate or deficient diet *(n = 4)*.

Fatty Acid Content (weight %, mean ± SD)		
	Adequate	Deficient
16:0	18.4±1.1	18.0±0.8
18:0	20.6±0.7	20.3±0.5
20:0	0.6±0.1	0.8±0.1
22:0	0.6±0.2	0.8±0.3
24:0	1.6±0.5	1.7±0.4
16:1	0.6±0.1	0.5±0.03*
18:1*n9*	20.4±1.7	19.0±1.0
18:1*n7*	5.0±0.2	5.6±0.2*
20:1*n9*	3.8±0.9	4.3±0.6
24:1	2.7±0.8	3.2±0.5
18:2*n6*	0.6±0.1	0.4±0.08*
20:3*n6*	2.2±0.3	2.2±0.2
20:4*n6*	6.2±0.4	8.1±0.5**
22:4*n6*	1.5±0.3	3.4±0.3***
22:5*n6*	0.0	7.7±0.7***
22:6*n3*	15.1±2.1	3.9±1.0***

* p<0.05, **p<0.01 and ***p<0.001 compared to the adequate diet group.

### Effects of Brain DHA Status on Motor Function Recovery after TBI

**Figure 1:**
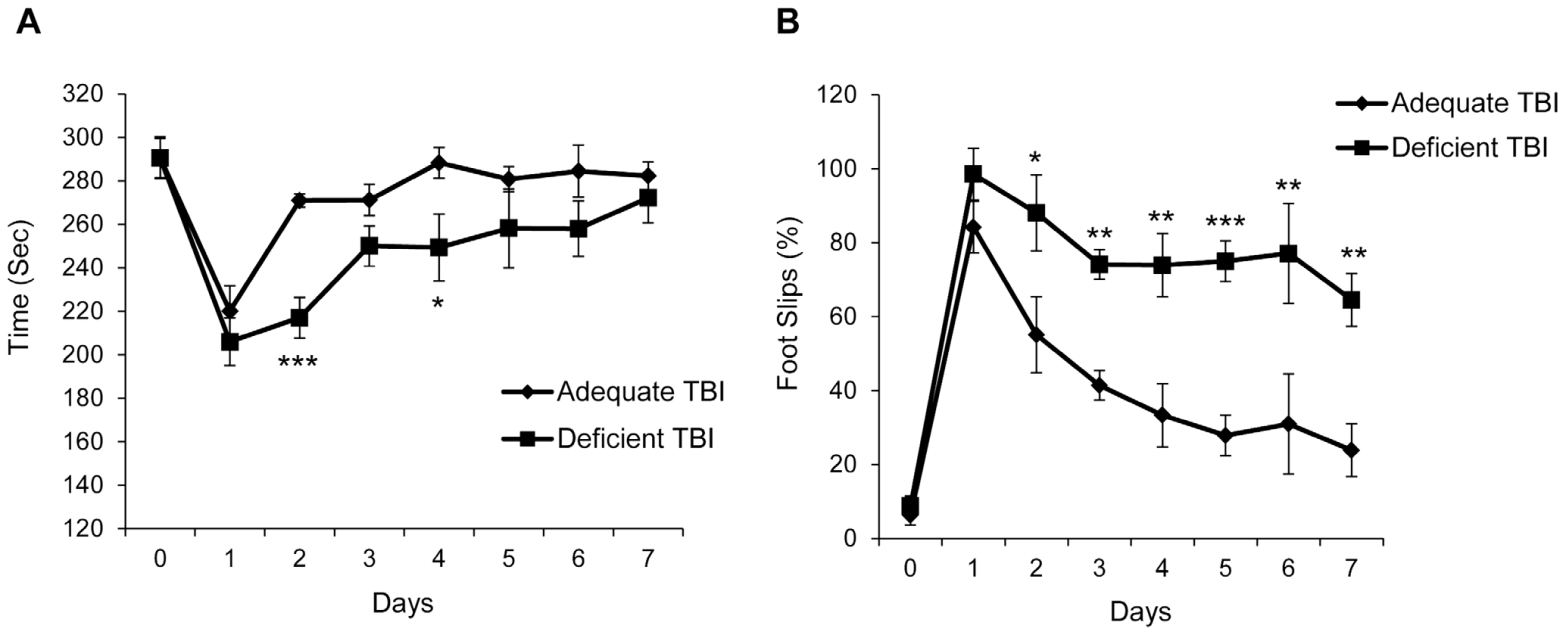
Omega-3 fatty acid deficiency impairs recovery from TBI-induced motor deficits. (A) The rotarod test showing slower recovery from TBI in the DHA deficient group (Deficient TBI) as compared to the adequate (Adequate TBI) mice with statistically significant differences on day 2 and day 4 after TBI (* *p*<0.05 and *** *p*<0.001 vs. the respective O-3 adequate group; *n = *8). (B) The beam walk test showing greater hindlimb footslips in DHA deficient mice as compared to the respective adequate controls (* *p*<0.05, ** *p*<0.01 and *** *p*<0.001 vs. adequate TBI group; *n = *7–8).

There was a significant difference in the latency to fall off the rotarod between the two diet groups of mice after TBI (Figure 1A). The DHA deficient mice showed delayed recovery from the day 1 to day 7 after TBI (*p*<0.01) as calculated by repeated measures Two way ANOVA. Further analysis by applying Sidak’s multiple comparisons test revealed significant differences on day 2 (adjusted *p*<0.001) and day 4 (adjusted *p*<0.05). The two diet groups had virtually identical latency periods (290.6 ±3.2 for adequate vs. 290.5±4.1 for deficient) when tested after three days of training (on the day before surgery), indicating that DHA depletion in the brain did not result in performance deficits in motor skills in naïve mice. The foot slips during the beam walk test were converted to percent of total steps and the data were analyzed by Two-way ANOVA. A highly significant difference was found between the two diet groups (*p*<0.0001) with the DHA deficient group showing more foot slips (Figure 1B). Sidak’s test for multiple comparisons revealed statistical significance at all time points from Day 2 through Day 7 after surgery except for Day 3. The number of foot slips by both groups was virtually identical on the day before surgery (6.3±2.7 and 8.8±2.2 respectively).

### Effects of Brain DHA Status on Anxiety-like Behavior and Cognitive Deficits after TBI

**Figure 2:**
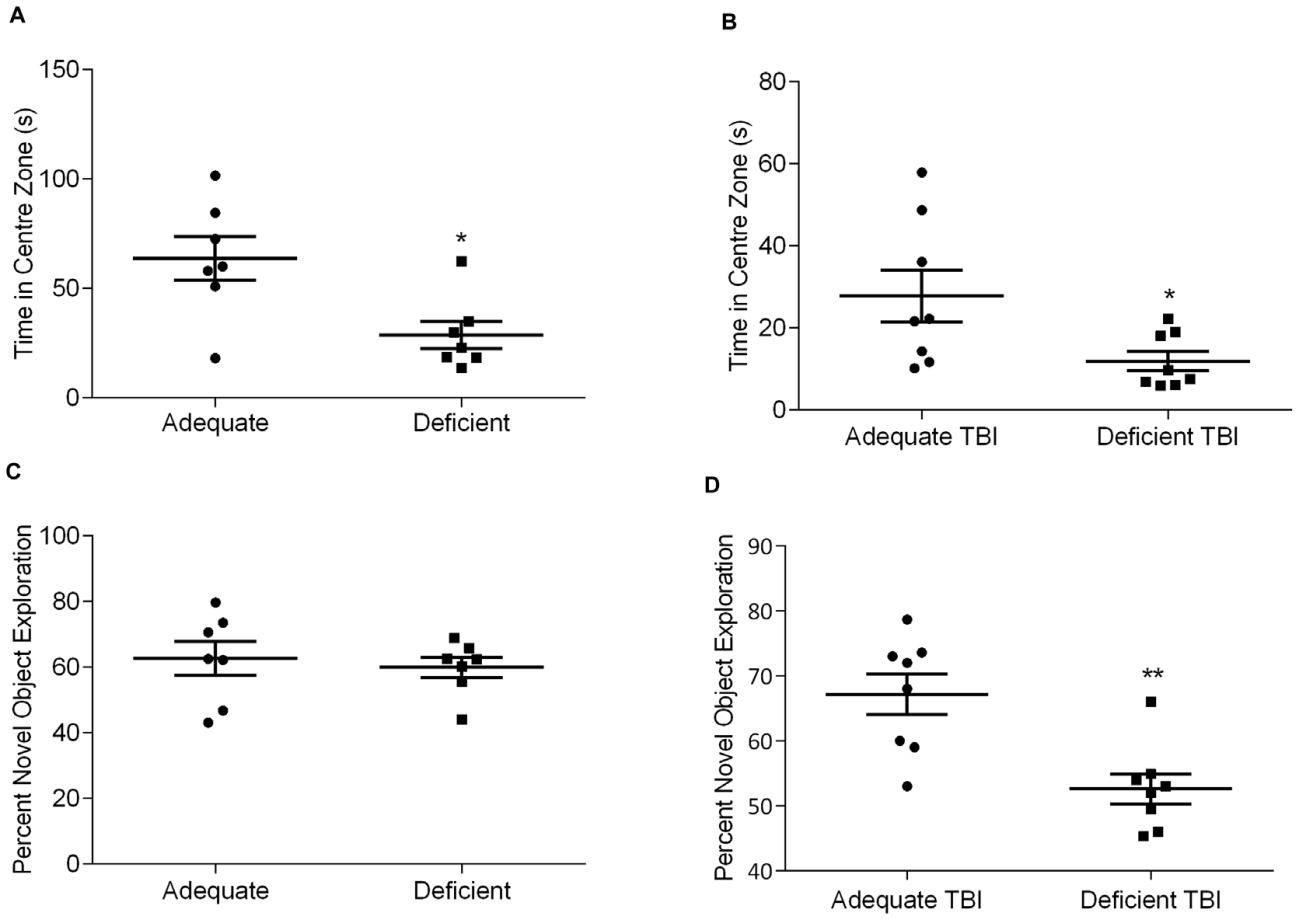
DHA depleted mice exhibit greater anxiety-like behavior and post-TBI memory deficits. (A) DHA deficient (Deficient) mice spend significantly less time in the centre zone of the open field than their DHA adequate (Adequate) counterparts (* *p*<0.05 vs. Adequate), thus exhibiting anxiety-like behavior. (B) A similar pattern of exploration observed on day 5 after TBI (* *p*<0.05 vs. Adequate TBI; *n = *8). (C) No significant difference observed in novel object exploration time between the non-injured DHA adequate and deficient mice (*n = *7). (D) Significantly less exploration of the novel objects in DHA depleted mice on day 7 after TBI than their adequate counterparts (** *p*<0.01 vs. Adequate TBI; *n = *8).

Anxiety-like behavior was studied by using the open field test and cognitive ability was assessed by the novel object recognition test. The time spent in the centre zone of an open field arena was used as to assess anxiety-like behavior. An unpaired two-tailed t-test yielded significant difference between the two diet groups, indicating that severe DHA deficiency leads to more anxiety-like behavior (63.7±10.0 vs. 22.9±4.3 for the omega-3 adequate group, *p*<0.05) (Figure 2A). This test was repeated for the TBI diet groups, which had similar differences in the time spent in the centre zone (28.7±6.2 vs. 11.1±3.1 for the DHA deficient groups, *p*<0.05) (Figure 2B).

The brain injured mice on omega-3 adequate diet had greater novel object exploration score than their deficient counterparts with a highly significant difference between the means (67.2±3.1 vs. 52.6±2.3 p<0.01) (Figure 2D). The non-injured mice did not show any difference between the two diet groups in the exploration time of the novel object, indicating that with our settings, the diets alone did not affect the ability of the mice to discriminate between familiar and novel objects (Figure 2C).

### Spectrin-αII Breakdown and Synapsin-1 Expression

**Figure 3:**
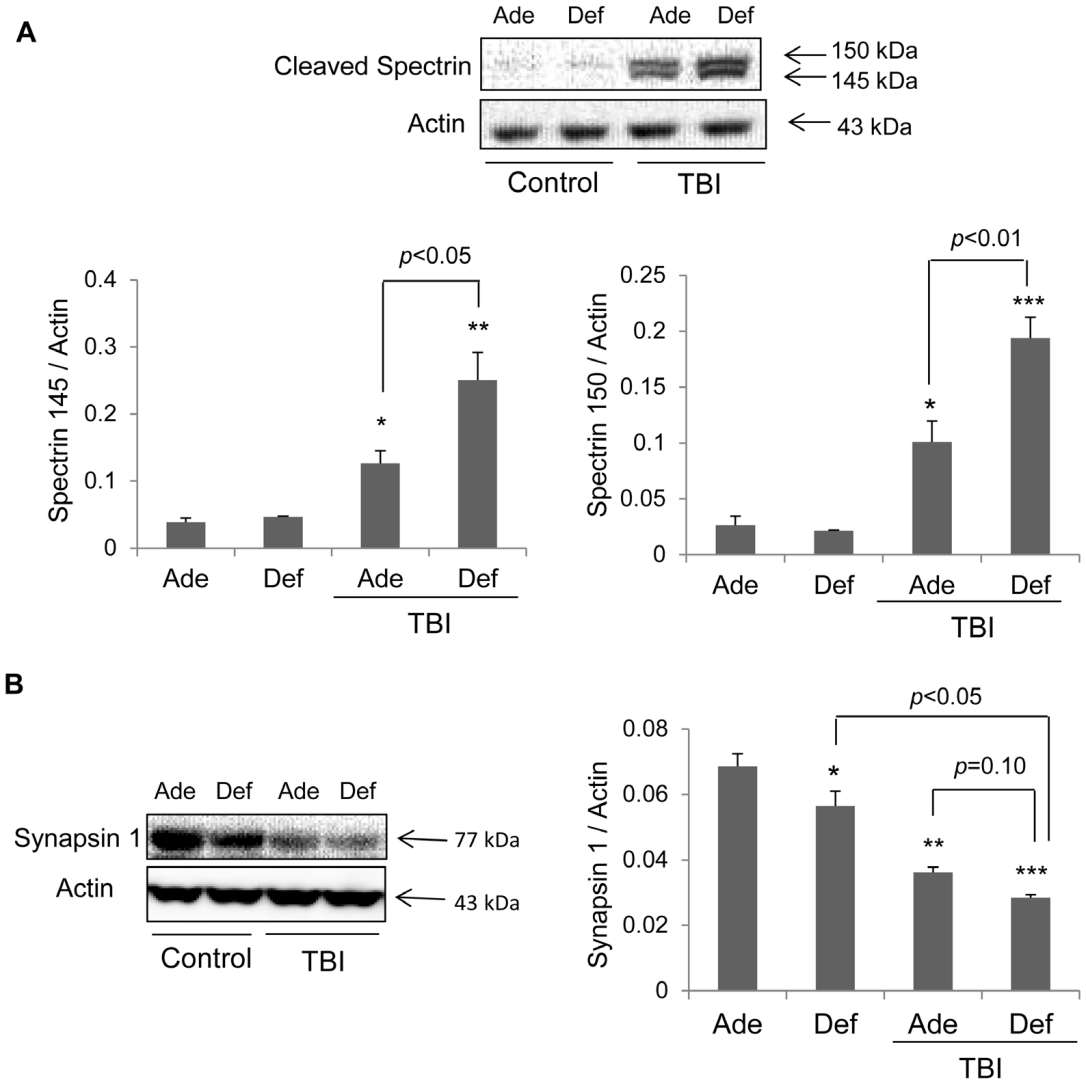
DHA deficiency increases TBI-induced spectrin-αII breakdown products (SBDPs) and decreases synapsin 1. (A) SBDP levels differentially increased after TBI in the affected cortices of DHA adequate (Ade) and deficient mice (Def). The contralateral cortices were used as controls. The deficient TBI group showed a significantly greater increase in SBDP levels after TBI for both 145 kDa and 150 kDa fragments as compared to the adequate TBI group (*n = *4*)*. (B) Synapsin 1 level affected by TBI and DHA depletion. TBI significantly decreased the synapsin-1 level in both diet groups. DHA depletion lowered the synapsin significantly (*p*<0.05) in the non-injured mouse cortices. Although statistical significance was not reached, a trend of further decrease in synapsin 1 level after TBI was observed with DHA deficiency. * *p*<0.05; ** *p*<0.01; *** *p*<0.001 compared to the non-injured adequate group.

TBI-induced breakdown of alpha-spectrin or alpha-fodrin, a marker of TBI, was assessed by western blotting (Figure 3A). Spectrin breakdown products (SBDPs) were detected at 145 kDa and 150 kDa. Densitometric analysis indicated that TBI significantly increases SBDPs in both diet groups. The cerebral cortex from the adequate animals had more than 3 fold increased levels of SBDP after TBI (*p*<0.05) and the DHA depleted group showed an even greater (more than 6 fold) elevation (*p*<0.01 for 145 kDa product; *p*<0.001 for 150 kDa product). Consequently, injured cortices of the DHA deficient mice had a relatively higher increase of both 150 and 145 kDa fragments as compared to those of the adequate diet group (*p*<0.05) (Figure 3A). Synapsin-1 protein expression was about 30% less in the cortex of the contralateral hemisphere of the mice fed with omega-3 fatty acid deficient diet (*p*<0.05) compared to those on adequate diet, indicating that DHA deficiency is by itself sufficient to decrease synapsin-1 expression as reported earlier [25]. TBI resulted in the reduction of synapsin 1 in both diet groups with the synapsin level in the adequate and deficient groups at 60% (p<0.001) and 45% (*p*<0.001) of the respective controls. Despite the lack of statistical significance in the differences in synapsin 1 level between the injured diet groups, a decreasing trend was observed in the deficient TBI group (Figure 3B).

### NeuN Immunostaining

**Figure 4:**
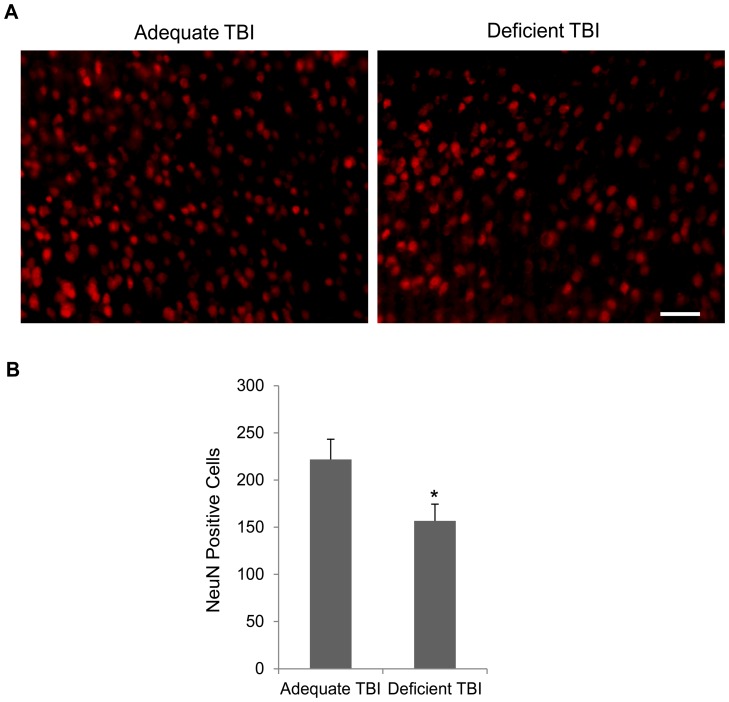
DHA deficiency decreases NeuN positive cells after TBI. DHA depleted mice showed decreased NeuN positive cells in the peri-contusional cortices relative to the DHA adequate mice as indicated by the representative micrographs (A) and quantitative analysis (B) of 25 brain sections from 5 mice. Scale bar  =  50µm. * *p*<0.05.

Immunostaining with NeuN and subsequent quantification indicated substantial decrease in NeuN-positive cells in the peri-contusional area. The margins of the injured area were mostly devoid of specific staining and the NeuN staining gradually increased away from the injury site. The deficient group showed reduced NeuN positive cells (157.0±17.8) in comparison to the adequate group (222.0±21.4) (*p*<0.05) (Figure 4).

### TBI Lesion volume

**Figure 5:**
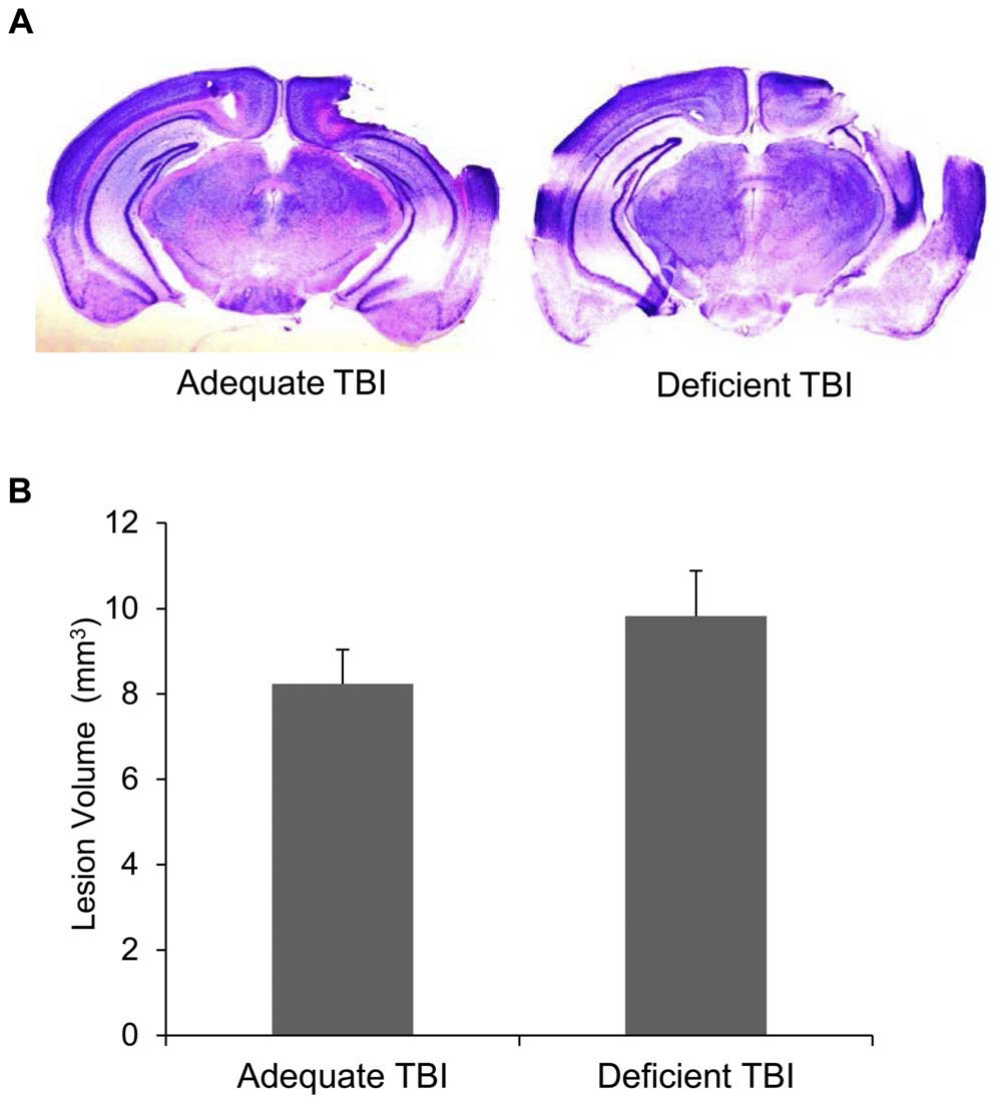
Depletion of DHA does not affect the volume of the lesion induced by TBI. Lesion volumes of DHA depleted brains after TBI are not statistically different from those of the DHA adequate controls (*n* = 6).

TBI by controlled cortical impact resulted in distinct cavitation in the injured cerebral hemisphere (Figure 5A). Although the DHA-depleted animals showed an increasing trend of the lesion volume (8.24 mm3±0.8 SEM and 9.83±1.06 SEM for DHA adequate and DHA depleted TBI mice respectively), the difference was not statistically significant (Figure 5B).

## Discussion

The present study demonstrates that i) severe DHA deficiency in the brain impairs functional recovery from TBI in terms of vestibulo-motor and cognitive deficits (ii) DHA deficiency further elevates TBI-induced production of SBDPs (iii) less neurons were found around the injury site of DHA deficient brain after TBI compared to the omega-3 fatty acid adequate group. Little is known regarding the effect of brain omega-3 fatty acid depletion on the predisposition toward altered recovery from TBI. Studies report that omega-3 fatty acid deficiency for a single generation can only decrease the DHA content by up to 40% whereas severe DHA depletion (up to 80%) can be achieved after two or more consecutive generations [26]. Over 70% of DHA depletion was observed in the cerebellum by feeding animals with omega-3 fatty acid deficient diet for three generations in the present study. It should be noted that the fatty acid analysis was performed for cerebellum, a part that has a vital role in the control and coordination of vestibulo-motor activity. We presume that the change in DHA levels in the cortex would be similar to that in the cerebellum. Indeed, the change in DHA level in the cerebellum in rats reared on omega-3 fatty acid deficient diet for two successive generations is comparable to that in the cortex [27]. Improved motor performance has been observed after focal cerebral ischemia [14] and neonatal hypoxic-ischemic brain injury [28] and spinal cord injury [29] after treatment with omega-3 fatty acids and in peripheral nerve injury [30] in fat-1 transgenic mice that have elevated endogenous omega-3 fatty acid level. The present study shows an improvement in the vestibulo-motor function in TBI mice by employing two motor tests. The rotarod test, particularly the accelerated version of the rotarod test, is a sensitive test to evaluate TBI induced motor deficits [19]. Our results indicate that recovery from acute motor deficits is impeded in the DHA deficient mice, which have shorter latencies on the rotarod. The beam walk test has been extensively used to detect differences in fine motor coordination. The difference in spontaneous recovery assessed by this test is even more pronounced than that evaluated by the rotarod test with the deficient group having more than 50% foot slips after a week from injury. These findings indicate that DHA deficiency interferes with the recovery of vestibulo-motor function after trauma. Incidentally, two studies were published during the preparation of this manuscript that deal with the effect of supplementation or depletion of omega-3 fatty acids/DHA on the outcome after cortical impact injury. Pu et al. have found improved functional recovery and reduced white matter damage after cortical impact injury in mice supplemented with omega-3 fatty acids for two months [31]. Similarly, in a study employing age-specific rat juvenile TBI model, Russell et al. found worsened motor deficits after TBI in DHA-depleted juvenile rats as compared to those on the control diet [32]. This is consistent with our findings in that DHA depletion in the brain by chronic omega-3 fatty acid deficiency exacerbates the injury and impedes the functional recovery.

The effect of deficiency of omega-3 fatty acids on anxiety and memory impairment has been extensively studied. There have been mixed reports regarding the effect of omega-3 deficiency on anxiety. Takeuchi et al. found that omega-3 deficient rats exhibit greater anxiety-like behavior in the elevated plus maze that was reversed by DHA supplementation [33]. In contrast, no significant differences were found by Nakashima and colleagues [34]. Carrie et al. showed that although omega-3 deficient mice showed greater anxiety in the open field, it was only partially alleviated on reversing the deficiency [35]. We found significant decrease in the time spent in the centre zone by DHA deficient mice for both sham and TBI groups indicating that the basal anxiety differences are due to DHA depletion and are maintained after trauma.

Many studies have demonstrated decrease in cognitive functions as a result of omega-3 fatty acid deficiency created through differential dietary regimens. In an experimental setup similar to the present study, Moriguchi et al. have reported that omega-3 fatty acid deficiency has a “dose dependent” impact on spatial memory impairment assessed by the Morris water maze test with the third consecutive generation of rats reared on omega-3 deficient diet showing greater deficits in memory as compared to the second generation [24]. Moreover, in a subsequent paper, the authors reported that such omega-3 fatty acid deficiency induced memory impairment is reversible and can be corrected on omega-3 replenishment [36]. We did not find significant changes in object recognition memory in the sham diet groups (Figure 2C). This may be attributed to two causes. One reason may be that the novel object recognition test assesses non-spatial object recognition. The second possible reason is that the duration of training for the novel object recognition test in the present study may be long enough to conceal any subtle cognitive deficits due to DHA depletion alone. Nevertheless, the changes in percent exploration of the novel object after injury are significantly different between the diet groups (Figure 2D). Also, for the mice on omega-3 fatty acid adequate diet, the percent novel object exploration score of the sham mice is comparable to that of the TBI mice. We believe that the main reason for this is the long exploration time during the training phase, which was up to 15 min, coupled to a shorter interval before the test phase, as opposed to the shorter training durations of 5 min generally used for this paradigm [37]. Thus the inability of the DHA deficient TBI mice to recognize the novel object despite the extended training emphasizes compromise in cognition in this group. Furthermore, DHA in the adequate diet, even though it was present in low amount, may also contribute to protection from cognitive impairment after TBI.

Membrane DHA in the brain provides resilience against propagation of injury-induced cellular damage and facilitates adaptive responses for recovery that are not adequately compensated for by omega-6 DPA that replaces DHA in the brains of mice on omega-3 fatty acid deficient diet. Indeed, naïve rats that were artificially hand fed with linoleic acid supplemented with DPA during infancy and later with similar pellets had correspondingly lower brain DHA levels and defective spatial retention as compared to the DHA-supplemented rats [38]. It is also possible that the increase in free fatty acids due to activation of phospholipases after TBI is a contributing factor in promoting recovery. A localized increase in free fatty acids at the site of injury has already been reported [39], [40]. In this case, DHA deficient mice would be expected to release less free DHA that can be converted to neuroprotective, anti-inflammatory and proresolving mediators such as neuroprotectin D1 or resolvins [41], which may result in increased susceptibility of neurons and greater neuronal loss encountered in this study. In addition, low free DHA would limit the production of synaptamide (docosahexaenoylethanolamide), a neurogenic, neuritogenic and synaptogenic metabolite of DHA [42], [43] that may have a bearing on functional recovery from trauma.

Non-erythroid alpha-spectrin II is a neuronal cytoskeletal protein that is a substrate for calcium activated cystein proteases, calpains and caspase-3. 145 kDa and 150 kDa fragments of alpha-spectrin II are remain elevated after severe TBI for 48 hr [44]. The breakdown products of alpha-spectrin II have been proposed as a reliable marker for brain injury [45] and to predict the severity of injury in severe TBI [46]. In this study, TBI in the DHA deficient mice resulted in greater cortical SBDPs, implying more tissue damage occurred in the deficient brain. Also, increased spectrin break down may be interpreted as increased activation of these cystein proteases after injury in the omega-3 deficient mice. Wu et al. found that DHA supplementation in diet alleviates the decrease in CAMKII, a substrate of calpains, due to fluid percussion injury [11]. It is possible that omega-3 fatty acids may be able to modulate injury processes by regulating calcium activated pathways. Indeed, omega-3 fatty acids have been reported to inhibit native T-type calcium ion channels [47]. Synapsin 1, a phosphoprotein enriched in synapses and implicated in synaptogenesis [48], was found to be significantly reduced after TBI in both DHA adequate and deficient mice, suggesting that TBI induces loss of synapses. The decreasing trend of the synapsin level in deficient brains also suggests a role of DHA in sustaining synapses after TBI.

Membrane changes due to DHA depletion may result in increased susceptibility of neurons to cell death after injury, thereby leading to greater decrease in NeuN positive cells in these mice. DHA as a membrane component has been reported to be a positive modulator of neuronal survival by promoting Akt mediated survival signaling [49]. Chronic dietary DHA modulation decreases neuronal damage by decreasing post-ischemic inflammation [50]. The decrease in the number of NeuN positive cells after TBI in this study is likely to involve similar mechanisms. However, the decrease in brain DHA level did not reflect as increase in tissue loss after TBI. The lack of statistically significant change in the frank lesion volume may be because the gross estimation of lesion volume did not have adequate sensitivity to detect small differences coupled with modest sample number used for this analysis. However, these results are similar to Pu and colleagues’ findings of relative increase in neurons in hippocampal CA3 in the omega-3 fatty acid (DHA/EPA) supplemented TBI mice without any change in the lesion volume [31] and also to Russell et al.’s finding of lack of change in lesion volume in DHA depleted juvenile rats after TBI [32]. Thus, although DHA/omega-3 fatty acids seem to effectively reduce infarction after cerebral ischemia [14], [15], [28], they may not affect frank lesion volume after TBI [31], [32].

In summary, severe depletion of membrane DHA in the brain renders mice significantly more susceptible to TBI and impairs recovery following the injury. Omega-3 fatty acids may serve as nutraceutical agents and precondition the brain to make it more resilient to injury. From this data, it can be suggested that enriching DHA in the brain may be prophylactic and protective against brain injury. Further studies with acute administration of DHA or its metabolites are needed to explore the possibility of its use at the therapeutic level.
